# Convergence and Divergence of Sugar and Cytokinin Signaling in Plant Development

**DOI:** 10.3390/ijms22031282

**Published:** 2021-01-28

**Authors:** Ming Wang, José Le Gourrierec, Fuchao Jiao, Sabine Demotes-Mainard, Maria-Dolores Perez-Garcia, Laurent Ogé, Latifa Hamama, Laurent Crespel, Jessica Bertheloot, Jingtang Chen, Philippe Grappin, Soulaiman Sakr

**Affiliations:** 1 Institut Agro, University of Angers INRAE, IRHS, SFR QUASAV, F-49000 Angers, France; ming.wang@qau.edu.cn (M.W.); jose.gentilhomme@univ-angers.fr (J.L.G.); sabine.demotes-mainard@inrae.fr (S.D.-M.); maria-dolores.perez-garcia@agrocampus-ouest.fr (M.-D.P.-G.); laurent.oge@agrocampus-ouest.fr (L.O.); latifa.hamama@agrocampus-ouest.fr (L.H.); laurent.crespel@agrocampus-ouest.fr (L.C.); jessica.bertheloot@inra.fr (J.B.); philippe.grappin@agrocampus-ouest.fr (P.G.); 2 College of Agronomy, Qingdao Agricultural University, Qingdao 266109, China; fuchao.jiao@qau.edu.cn (F.J.); chenjingtang@126.com (J.C.)

**Keywords:** nutrient, hormones, development, seeds, flowering, branching senescence, meristem, source–sink relationship

## Abstract

Plants adjust their growth and development through a sophisticated regulatory system integrating endogenous and exogenous cues. Many of them rely on intricate crosstalk between nutrients and hormones, an effective way of coupling nutritional and developmental information and ensuring plant survival. Sugars in their different forms such as sucrose, glucose, fructose and trehalose-6-P and the hormone family of cytokinins (CKs) are major regulators of the shoot and root functioning throughout the plant life cycle. While their individual roles have been extensively investigated, their combined effects have unexpectedly received little attention, resulting in many gaps in current knowledge. The present review provides an overview of the relationship between sugars and CKs signaling in the main developmental transition during the plant lifecycle, including seed development, germination, seedling establishment, root and shoot branching, leaf senescence, and flowering. These new insights highlight the diversity and the complexity of the crosstalk between sugars and CKs and raise several questions that will open onto further investigations of these regulation networks orchestrating plant growth and development.

## 1. Introduction

The regulation of plant growth and development is crucial for yield and resistance to abiotic and biotic constraints, which relies on fine-tuned interactions between nutrients and hormones, influenced by environmental inputs. Among these central regulators, sugars and cytokinins (CKs) play predominant roles while operating synergistically, antagonistically and sometimes independently to shape the final reaction of the plant. Sugars growth-related metabolic activity and as signaling entities that drive a wide array of mechanisms throughout the plant life cycle [1,2,3,4,5]. Briefly, sugar signaling is intimately linked to developmental stages, hormonal signaling and environmental conditions, and thereby is an integrative part of plant growth control [6,7,8,9,10,11]. Plants can sense a diversity of soluble sugars such as sucrose, glucose, fructose and trehalose-6-phosphate (T6P). Sophisticated sugar sensing networks have been identified, including hexokinase (HXK), Regulator of G-protein signaling (RGS1), and two main sensors of nutrients and energy status: sucrose-nonfermentation1-related protein kinase1 (SnRK1) and target of rapamycin (TOR) kinase [12,13,14,15,16,17,18].

CKs are a group of adenine derivatives involved in many central processes in plants, such as development of vasculature, differentiation of embryonic cells, maintenance of meristematic cells, shoot formation and leaf senescence delay [19,20,21,22,23]. There are two types of CKs: adenine-type cytokinins represented by kinetin, zeatin, and 6-benzylaminopurine, and phenylurea-type cytokinins like diphenylurea and thidiazuron. Most adenine-type cytokinins are synthesized in roots. Cambium and other actively dividing tissues also synthesize CKs. CKs are viewed as one of the major long-distance root-to-shoot messengers [24]. Their biosynthesis depends on the activity of adenosine phosphate-isopentenyltransferases (IPTs). Trans-zeatin is the most abundant form of CK in plants [25]. Initially identified in rice, Lonely Guy (LOG), cytokinin nucleoside 54-monophosphate phosphoribohydrolases, are involved in direct CK production [26,27]. CKs primarily regulate gene expression through a phosphotransfer signaling cascade. This cascade is initiated by histidine kinase cytokinin receptors, Arabidopsis Histidine Kinase2 (AHK2), AHK3 and AHK4, that located in the endoplasmic reticulum membrane, and completed by cytosolic histidine phosphotransfer proteins (AHP) [28]. AHPs shuttle between the cytosol and the nucleus and transfer phosphate to nuclear response regulators (Arabidopsis Response Regulators, ARRs) [19,23] that fall into two classes: type-A and type-B ARRs are negative and positive regulators of CK signaling, respectively.

**Figure 1:**
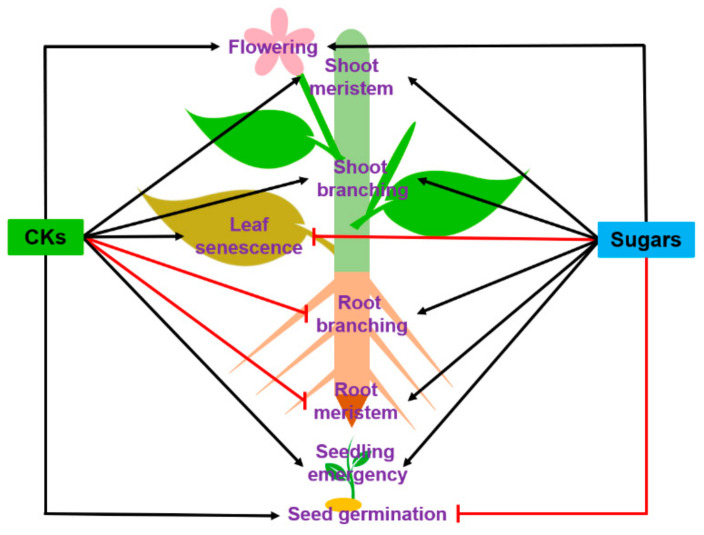
Relationship between sugars and cytokinins (CKs) in the main plant developmental processes, including seed development, germination, seedling establishment, root and shoot branching, leaf senescence, and flowering. The black arrows indicate stimulation or positive effect, and the red lines mean repression or negative effect. This model results from a compilation of studies carried out on different model plants (see references and description in the text).

Sugars and CKs are individually viewed as major players in many aspects of plant biology. Yet, their crosstalk has not been systematically investigated, hence many gaps in current knowledge. Moreover, the available results underline that the crosstalk is very complex and varies at least according to the nature of the organ and the physiological process. This review aims to underline the interactions between sugars and CKs based on their individual and combined roles in the regulation of key developmental processes throughout the plant life cycle. Based on the results derived from different plant species, sugars and CKs seem to act synergistically to take over the seedling emergency, shoot meristem activity, shoot branching and flowering while doing antagonistically as strongly suggested for seed germination, root meristematic activity, and even demonstrated for root branching and leaf senescence (Figure 1). Here, the main results are discussed, potential integrators of this crosstalk are proposed, and further lines of research are highlighted.

## 2. Seed Development, Germination and Seedling Establishment

Seed formation, as well as the seed-to-young-seedling transition through germination, involves sugar and hormone signaling [29,30]. Even though common key players have been identified in the seed response to sugars and CKs, their molecular interaction remains speculative.

### 2.1. Seed Development

Seed development covers morphogenesis phases characterized by active cell division and embryonic organ formation and a maturation phase during which storage nutrients accumulate in cotyledons and/or endosperm tissues, with a transfer of reserves between these two compartments [31]. In this latter phase, the embryo acquires tolerance to desiccation and a dormancy state before dispersal in the environment. Dormancy allows the seed to cope with its adverse environment and secures the transition to a new life cycle. Previous works have reported the contribution of sugars and CKs in the control of seed development [32,33]. In cotyledons of *Vicia faba*, a high glucose-to-sucrose ratio is correlated with cell division during the morphogenesis phase, whereas an increasing sucrose-to-glucose ratio marks the sink–source transition to the storage phase [34]. The high glucose gradient is related to both high cell-wall-bound invertase (*CWINV*) expression in the maternal seed coat and hexose transporter (*VfSTP1*) expression in the embryonic epidermal cells [35,36]. Analyses of the *CWINV*-deficient mutant *miniature1* (*mn1*), impaired in endosperm development in maize caryopses, provide evidence that *CWINV* also contributes to CK-dependent cell proliferation during the developmental transition to the storage phase [37,38,39]. Such a CK effect may operate directly on cell cycle-related genes (*CycD3*) and indirectly through (*CWINV2*)-mediated sugar signaling [37,40,41]. Nevertheless, the seemingly contradictory phenotype of the CK-receptor-defective triple mutant *ahk2 ahk3 cre1* exhibiting greater seed size points to the complexity of the regulatory network [42]. Understanding how CKs contribute to seed development will require considering the different levels of regulation of CK metabolisms, such as the spatiotemporal accumulation and transport of CKs in seed tissues, the dynamics of their biosynthesis (IPT) and inactivation (CKX), and their perception. The transition from cell division and expansion (seed morphogenesis) to storage activity (seed maturation phase) is associated with downregulated *CWINV* and *IPT* expression [43,44]. At this stage, sugars serve for seed storage accumulation by mediating sucrose synthase induction for starch biosynthesis in maize kernels [33,45] or gibberellic acid (GA) dependent α-amylase induction for storage remobilization in barley embryos [46]. Such sugar-dependent regulation takes place at the transcriptional and post-transcriptional levels [2]. The role of sugars in seed maturation could be complex and partially mediated through T6P, considered as a proxy for sucrose availability in plants [47], and SnRK1 [48]. Sucrose positively regulates T6P accumulation in wheat at the seed pre-filling stage [49], and its exogenous application stimulates seed filling and yield [50]. Accordingly, *Arabidopsis* seeds of the mutant *tps1* (Trehalose-6-phosphate synthase 1) fail to proceed towards the maturation phase [51,52]. In pea, SnRK1 deficiency hinders the maturation and storage activity [53,54]. Accordingly, SnRK1 induces abscisic acid (ABA) synthesis and signaling and the C/S1-group bZIP signaling pathways associated with carbon starvation [55,56]. This regulation is mediated by pFUS3 (The *Arabidopsis* B3-domain transcription factor FUSCA3) phosphorylation, known to control ABA responses during seed maturation and dormancy [57]. Transcriptomic comparison of CK metabolism and signaling in dormant and non-dormant wheat seeds [58,59] highlights that CK controls the activity of many genes involved in seed dormancy. The interactions of CKs with ABA metabolism and signaling during seed maturation need to be further investigated and compared with sugar signaling mediated at least by the T6P and SnRK1 pathways.

### 2.2. Seed Germination and Seedling Establishment

**Figure 2:**
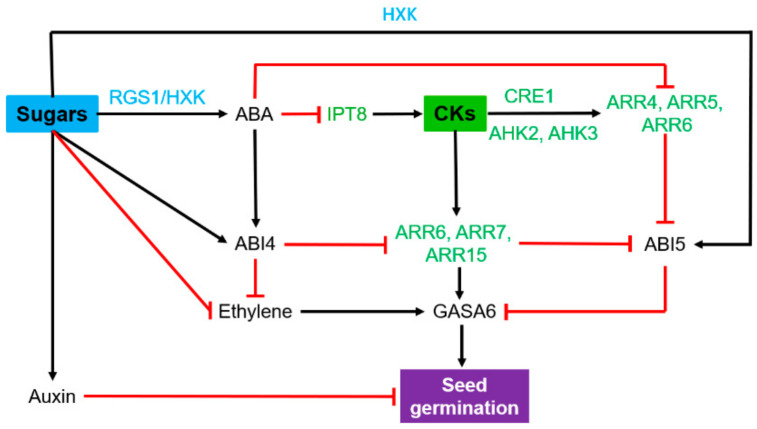
Antagonistic effect of sugars and cytokinins (CKs) on seed germination. Blue stands for players of sugar signaling pathways, and green highlights genes involved in CK synthesis or signaling pathways. Black arrows and red lines indicate stimulatory and inhibitory effects, respectively. ABI, abscisic acid insensitive; AHK, *Arabidopsis* histidine kinase; ARR, *Arabidopsis* response regulator; CRE, cytokinin response; GASA, gibberellic acid-stimulated *Arabidopsis*; HXK, hexokinase, IPT: isopentenyl transferase; RGS, regulator of G-protein signaling. This model results from a compilation of studies carried out on different model plants (see references and description in the text).

The carbon stored in the mature seed will be remobilized during germination to ensure seedling establishment before becoming heterotrophic. Seed germination is accomplished when the radicle protrudes through the outer layers of the embryo, i.e., the endosperm and the teguments [60]. The related cellular and metabolic events are orchestrated by complex signaling crosstalk involving the hormones ABA and GA, well known for their role in inhibiting and inducing germination, respectively [61]. Sugars released from the GA-mediated hydrolysis of storage compounds and cell wall loosening serve as osmotically active solutes for radicle cell expansion. These sugars are potentially used as central signals of the seed’s C status and are also a source of C for seedling growth during the transition to autotrophy. Genetic and molecular analysis revealed a possible control of germination by glucose based on HXK1-dependent and independent pathways and the T6P pathway, interacting with different hormonal pathways [29]. Many reports also showed that CKs contribute to the control of seed germination [42]. However, their interactions with glucose are poorly documented. On the whole, glucose and CKs are likely to operate antagonistically at different steps of the ABA biosynthesis and signaling pathways (Figure 2). The contribution of glucose to the control of seed germination has long been established and proven to be a concentration-dependent signal [62,63,64]. Exogenous supply of high glucose contents delays seed germination through positive regulation of ABA synthesis, accumulation and signaling [65,66,67,68]. At lower concentrations, glucose stimulates germination by inducing ABA catabolism [69]. In germinating seeds, high glucose supply upregulates two ABA biosynthesis genes (*NCED3* and *ABA2*) through the G Protein Alpha subunit AtGPA1 and the Regulator of G-protein Signaling AtRGS1, via an HXK1-independent channel [70,71,72]. Glucose also repressed—the positive regulator of seed germination AtGASA6 via an HXK1-dependent pathway [73,74]. AtGASA6 acts as an integrator of ABI5-dependent ABA signaling and RGL2-dependent GA signaling [73]. Therefore, a high level of T6P promotes seed germination by decreasing seed sensitivity to glucose and ABA [75,76,77]. In sum, the inhibition of seed germination under excessive glucose supply conditions may be due to the activation of the ABA signaling pathway and an imbalance in sugars/T6P [56].

The CKs are described to stimulate seed germination by an antagonistic effect on ABA signaling [78,79,80]. In germinating seeds, increasing levels of CKs induce the expression of type-A ARRs (ARR4, ARR5 and ARR6) that inactivate the ABI5-mediated inhibition of germination [81,82] whereas glucose enhances ABI5 transcription [83] (Figure 2). In turn, ABA intake represses CK biosynthetic genes such as *AtIPT8* and CK signaling genes such as type-A ARRs, and during seed dormancy, ABA signaling, including ABA receptor Pyrabactin Resistance (PYR/PYL), SnRK2s and ABI4, downregulates type-A ARRs [84]. In dormant seeds, high ABA levels positively regulate ABI4, which inhibits the expression of *ARR6*, *ARR7* and *ARR15*. Either, *Arabidopsis* CK-receptor mutants exhibit a reduced dormancy phenotype, and distinct CK-mediated seed germination regulation pathways seem to exist [42]. In germinating seeds, many other regulatory pathways respond to different forms of sugar signals. The exogenous sugar-dependent inhibition of seed germination is also regulated by the sucrose transporter SUT4/Cyb5-2-mediated signaling pathway, independently of the ABA (ABI2/ABI4/ABI5)-mediated signaling pathway [85]. CK biosynthesis is noticeably concomitant with SUT gene expression during pea seed germination. Therefore, we may wonder whether sugar transporters could be a convergent target of sugars and CKs during this process [86].

Interestingly, promoters of the senescence-associated genes *SAG12* and *SAG13* are inducible in the tomato seed micropylar endosperm [87], suggesting that a senescing mechanism known to be stimulated by HXK1-dependent sugar signaling (see Leaf Senescence Section 5) could facilitate radicle protrusion. Ectopic expression of the *IPT* gene through *SAG12* and *SAG13* promoters delayed endosperm senescence and germination, suggesting that potential CK synthesis in the endosperm can antagonize the HXK-dependent sugar senescing mechanism to negatively control germination. Therefore, CKs could be perceived differently in a tissue-dependent manner during seed germination.

The crosstalk between sugars and CKs in the control of germination remains very partially documented, and available results foresee very intricate mechanisms. All the present results support antagonistic effects of glucose and CKs throughout the germination process, which precedes seedling growth considered as a post-germinative phase.

### 2.3. Seedling Development

Upon radicle protrusion through the seed coat, the first post-germinative events initiate seedling growth through hypocotyl elongation and root meristem development before the activation of the photosynthesis machinery. Hypocotyl elongation occurs in darkness and is fueled by C issued from the hydrolysis and mobilization of seed storage compounds. The shoot apical meristem (SAM) is characterized by a heterotrophic metabolism, while the development of the root apical meristem (RAM) occurs only under light conditions and is controlled by cotyledon-derived photosynthetic sucrose that acts as a long-distance signal [88].

CK and glucose signaling are involved in controlling different aspects of seedling growth and development, with auxin signaling components as downstream targets. From a physiological point of view, both glucose and CKs could control radicle growth in light conditions, hypocotyl length in darkness, chlorophyll and anthocyanin contents [89]. CKs interact with glucose via an HXK1-dependent pathway for the control of radicle and hypocotyl elongation [30,90]. SnRK1 overexpression can delay seed germination and increases sensitivity to glucose and ABA during seedling establishment [91]. When glucose is supplied to seedlings, T6P acts antagonistically to SnRK1 by inhibiting ABA synthesis and signaling and, in turn, the seed sensitivity to glucose [92]. Noteworthily, CKs antagonize ABA signaling by inhibiting SnRK2 activity via type-B ARRs and thus promote seedling establishment [93].

## 3. Meristem Establishment and Functioning

Sugars and CKs are fully part of the regulation of the dynamic balance between cell division and cell differentiation, which determines organ shape and size. Sugars can activate the expression of key cell cycle regulators, such as cyclin-dependent kinases (CDKs) and their interacting cyclins (CYCs), promoting the G2/mitosis transition in *Arabidopsis* seedling meristematic tissues [88,94,95,96,97]. CK signaling contributes to the stimulation of cell division and meristem initiation/formation [98,99].

### 3.1. Root Apical Meristems

**Figure 3:**
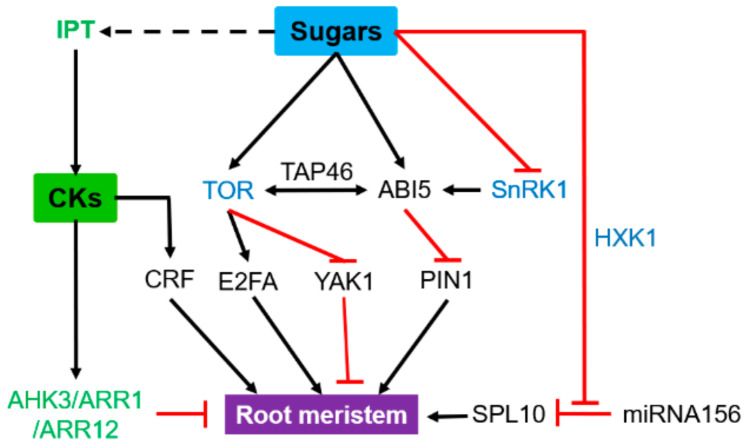
Antagonistic effect of sugars and cytokinins (CKs) on the functioning of the root meristem. Blue indicates sugar signaling pathways, and green highlights genes involved in CK synthesis or signaling pathways. Black arrows and red lines indicate stimulatory and inhibitory effects, respectively. Solid line, direct effect; dotted indirect effect. ABI5, abscisic acid insensitive 5; AHK3, *Arabidopsis* histidine kinase 3; ARR, *Arabidopsis* response regulator; CRF, cytokinin response factor; HXK1, hexokinase 1; E2FA, elongation 2 factor A; IPT, isopentenyl transferase; PIN1, PIN-FORMED 1; SnRK1, SNF1-related kinase1; SPL10, Squamosa Promoter Binding Protein-Like 10; TAP46, type 2A phosphatase associated protein of 46KD; TOR, target of rapamycin protein kinase; YAK1: Yet Another Kinase. This model results from a compilation of studies carried out on different model plants (see references and description in the text).

The root system consists of two principal root-types: the primary root (PR), which is formed embryonically and secondary roots, which form post-embryonically [100]. Glucose influence root meristematic activity through many pathways, including the macro-autophagy/autophagy degradation pathway, which acts downstream of SnRK1 and TOR kinase [101]. High concentrations of glucose reduce the size of the root meristem zone via ABI5, which represses the auxin efflux carrier PIN1 required for auxin accumulation in the meristem (Figure 3) [102]. Either, mounting evidence also indicates that SnRK1 and ABA can control root meristem activity cooper actively [103,104,105]. Overexpression of *SnRK1.1* results in an ABA-hypersensitive phenotype [104] due to its interaction with the regulator of ABA response PP2C phosphatase protein [106,107,108]. ABI5, the main node of the glucose and ABA pathways, is directly phosphorylated by SnRK1 [56,109,110]. Glucose induces *ABI5* expression, which reduces the size of the root meristem zone. *ABI5* can coordinate and adjust physiological and metabolic demands with growth, but also interact with TOR kinase—a highly conserved eukaryotic phosphatidylinositol-3-kinase-related kinase—through TAP46 (2A Phosphatase Associated Protein of 46 KD) to influence the ABI5 signaling pathway negatively [111,112,113]. TOR-kinase also plays a major role in the regulation of growth and metabolism in plants [114]. Glucose-driven TOR-kinase signaling regulates root meristem activation independently of hormonal and hexokinase signaling pathways and involves the upregulation of the elongation factor E2Fa [115]. In response to metabolic demands, the tonoplast sugar transporter (TST) imports sucrose, fructose and glucose into the root vacuoles to maintain cytosolic sugar homeostasis [116]. Yet Another Kinase (YAK) acts as a member of the dual-specificity tyrosine phosphorylation-regulated kinase and may be involved downstream of this TOR signaling-mediated control of root meristem activity in *Arabidopsis* [117].

CKs are essential to promote cell differentiation in the root meristem [118,119,120]. This is due to trans-zeatin, whose accumulation slows down the root growth rate and the cell transition to elongation, leading to prolonged mitotic cycles [121]. Mutants defective in all CK receptors display severely reduced sizes of their shoot and root meristems [122,123]. This CK-dependent reduction of the root meristem size could involve a two-component receptor histidine kinase and type A-ARR transcription factor, such as AHK3/ARR1, AHK3/ARR12, that regulates the rate of meristematic cell differentiation (Figure 3) [123,124,125]. The *Squamosa Promoter Binding Protein-Like* (*SPL*) transcription factor is one of the targets of microRNA156 (miRNA156). *miRNA156* and *SPL* have opposing expression patterns; high miRNA156 levels induce reduced root meristem size, while overexpression of *SPL10* increases it [126]. Furthermore, meristem activity is regulated by *SPL10*, probably through the reduction of CK responses via the modulation of type-B *Arabidopsis Response Regulator1* (*ARR1*) expression. This points to a cooperative regulation of root meristem activity by CK responses via miRNA156-targeted *SPL10* [126]. Given that miRNA156 is a central component of sugar signaling [127], it will be of high interest to investigate whether sugars could take part in this regulatory network. CKs also cooperate with other hormones to regulate root meristem development. In *Arabidopsis* roots, the *IAA3/Short Hypocotyl 2* (*SHY2*) gene is an important hub of the crosstalk between CKs, auxin and brassinosteroids (BRs) [128]. This calls for investigating its regulation by sugars. CK response factors (CRFs) are a group of related AP2/ERF transcription factors transcriptionally induced by CKs [129]. Overexpression of CRFs in *Arabidopsis* results in a larger root apical meristem. Disruption of CRFs was accompanied by low sensitivity to CKs in a root elongation assay, along with a reduced expression level of ARRs and of the homeobox gene *STIMPY* (STIP or WOX9) required for root and shoot apical meristem maintenance [19]. Although being acted antagonistically to regulate root meristem activity, additional investigations are required to bring the first mechanistic insights associated with molecular integrators involved in sugars and CKs crosstalk.

### 3.2. Shoot Meristem

**Figure 4:**
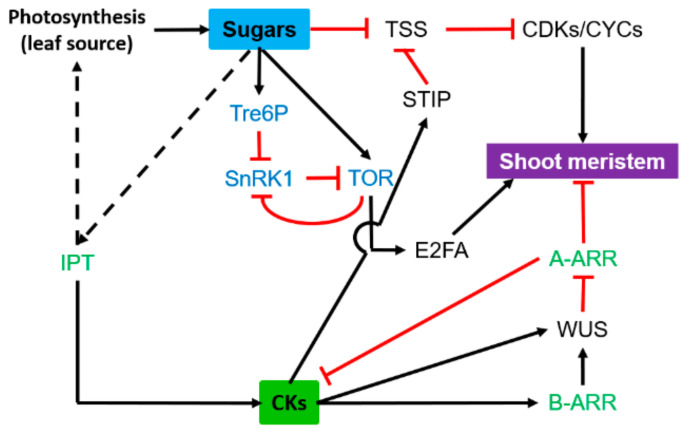
Synergetic effect of sugars and cytokinins (CKs) on the functioning of the shoot meristem. Blue indicates sugar signaling pathways, and green highlights genes involved in CK synthesis or signaling pathways. Black arrows and red lines indicate stimulatory and inhibitory effects, respectively. Solid line, direct effect; dotted indirect effect. A-ARR, type-A *Arabidopsis* response regulator; B-ARR, type-B *Arabidopsis* response regulator; CRF, cytokinin response factor; CDKs, cyclin-dependent kinases; CYCs, cyclins; E2FA, elongation 2 factor A; IPT, isopentenyl transferase; SnRK1, SNF1-related kinase 1; TOR, target of rapamycin protein kinase; Tre6P, trehalose-6-phosphate; TSS, TPR-DOMAIN SUPPRESSOR OF STIMP; WUS, WUSCHEL. This model results from a compilation of studies carried out on different model plants (see references and description in the text).

The shoot meristem contains a central zone (CZ) that harbors pluripotent stem cells and surrounding regions in which cells start to differentiate, and organ primordia are initiated. Sucrose and Glucose have long been known to promote meristem growth [1,130,131], and this effect could be mediated by the upregulation of CDKs and CYCs expression, which are required for the G1/S and G2/M transitions (Figure 4) [97,132]. While glucose signaling is sufficient to activate TOR kinase in root apexes, both glucose and light signals are required for TOR activation in shoot apices [133]. SnRK1 is expressed in the meristem and young leaf primordia; its low activity is required for CK biosynthesis [54,56,134], hence a link between nutrient/energy availability and CK production. In contrast with their role in roots, CKs promote shoot cell division through the regulation of a variety of key genes related to plant meristem activity and are essential to maintain undifferentiated cells [119,135,136]. The CLAVATA (CLV) ligand–receptor system and two transcription factors, SHOOTMERISTEM-LESS (STM) and WUSCHEL (WUS), are involved in meristem growth [137,138,139]. WUS, a positive regulator of stem cell proliferation, directly downregulates several type-A ARR transcription factors (ARR5, ARR6, ARR7 and ARR15), which act in the negative feedback loop of CK signaling (Figure 4) [140,141]. More interestingly, CK signaling precedes the de novo expression of *WUS* in the leaf axil to promote axillary meristem initiation via the direct binding of the type-B ARR transcription factor to the WUS promoter (Figure 4) [98]. Moreover, CK signaling can activate the meristem and maintain its fate by inducing *STIMPY* expression in meristematic tissues [142,143]. The IPT and LONELY GUY (LOG) genes, which encode a novel CK-activating enzyme operating in the final step of bioactive CK synthesis, are both pivotal for the conversion of CK hormone precursors into active hormones within the shoot meristem [26,136]. In line with this, CK deficiency mutants display low activity of the vegetative and floral shoot apical meristems [144]. However, an opposite effect has been reported in *Azolla* as compared to *Arabidopsis* [145].

Transcriptomic analysis identified that glucose could stimulate CK accumulation through the induction of *IPT3* expression and the repression of cytokinin oxidase (*CKX4*) and also control the expression of 76% of CK-regulated genes at the whole-genome level in *Arabidopsis* seedlings [90]. This study highlights that the interaction between glucose and CKs plays a key and synergistic role in shoot meristem activity. Additional work would be required to identify the main convergent node of the crosstalk between sugars and CKs. One approach would be to use the promoter of some common genes such as *CYCs*, *CDK*s to identify the upstream regulators.

## 4. Root and Shoot Branching

Plants comprise two distinct parts: (i) the shoot system for photosynthesis and reproductive functions, and (ii) the root system for water and nutrient uptake from the soil and anchorage. These two parts have evolved a complex branching strategy to increase their total surface area, ensuring a better adjustment of plants to their abiotic and biotic environments.

### 4.1. Lateral Root Growth

Shoot branches are formed by an actively dividing shoot meristem, whereas lateral roots are derived from the pericycle, located beyond the root subapical meristem zone [146]. Auxin regulates lateral roots (LRs) positioning, which determines the spatial distribution of lateral root primordia and LRs along with primary roots [147]. Auxin also regulates root outgrowth and LRs emergence through interaction with sugars and CKs. Sugars promote lateral root initiation using different pathways that are not always associated with auxin [148,149]. Map-based cloning revealed that a neutral invertase gene (*AtCYT-INV1*) had a significant influence on lateral root development by controlling the hexose concentration within cells [150]. Photosynthetically generated sugars induce *AtIPT3* and *CYP735A* expression to promote CK accumulation in roots [151]. This effect operates through the heterotrimeric G-protein complex (hexokinase-independent pathway), which regulates auxin distribution in the root and thereby induces lateral root formation [149]. More precisely, WOX7, a member of the WUSCHEL related homeobox (WOX) family of transcription factors, plays a major role in coupling lateral root development with the sugar status in plants [146]. WOX7 acts as a transcriptional repressor in lateral root development. Genetic, physiological, transcriptomic and grafting approaches evidenced that *C*-Terminally Encoded Peptide Receptor 1 (CEPR1) inhibited lateral root growth in response to sugars (including sucrose) and elevated light intensity [152].

CKs represses lateral root initiation and promote lateral root elongation [153,154,155]. They block cell cycling in the pericycle founder cells at the G2/M transition phase [156] and then disturb lateral root initiation in plants [157], confirming that the earliest stages of lateral root formation are very sensitive to the inhibitory effect of CKs [158]. Cytokinin Response Factor2 (CRF2), a component of the CK signaling pathway, plays an important role in regulating *Arabidopsis* lateral root initiation [159]. In *Arabidopsis* again, the mutation of *CYP735A* genes required for trans-zeatin biosynthesis causes strong defects in lateral root positioning, indicating a determining role for CK metabolites in the regulation of lateral root initiation [160].

The crosstalk between CKs and sugars also regulates root branching (Figure 4). Combined analysis in roots of grafted apple revealed that root growth and development of rootstocks were mainly influenced by the sugar metabolism, auxin, and CK signaling [161]. Furthermore, the crosstalk between glucose and CKs regulates root development in *Arabidopsis* [89]. These authors showed that CKs interact with glucose via an HXK1-dependent pathway for root length control. Wild-type (WT) roots cannot elongate without glucose, but roots elongate even in the absence of glucose in the CK-receptor mutant *Arabidopsis Histidine Kinase4* (*ahk4*) and type-B ARR triple mutant *arr1* (*Arabidopsis Response Regulator1*), *arr10*, *arr11* compared with the WT. Although 60 genes related to root growth are regulated by both CKs and glucose, nothing is known on the physiological relevance of the crosstalk between sugars and CKs in the control of root-lateral formation.

### 4.2. Shoot Branching

**Figure 5:**
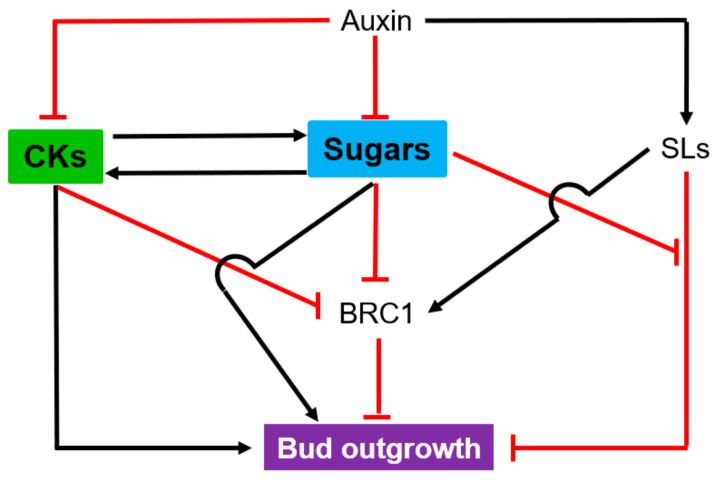
Synergistic effect of sugars and cytokinins (CKs) on bud outgrowth. Black arrows and red lines indicate stimulatory and inhibitory effects, respectively. SLs: strigolactones; BRC1: Branched1.

Shoot branching is a strictly regulated process that involves a very finely tuned hormonal and nutrient regulatory network [162,163,164,165] and is highly governed by environmental inputs [166]. In this intricate process, sugars and CKs behave as inducers, while auxin and strigolactones (SLs) act as repressors. However, whether sugars and CKs act synergistically or independently in this process remains unknown (Figure 5). Auxin derived from the young growing leaves of the apical meristem is transported down the stem through a specific polar auxin transport (PAT) stream and indirectly inhibits bud outgrowth through the opposite action of CKs and SLs [162,167]. The inducer effect of CKs has long been known [168]. This effect could occur through (i) the downregulation of the main inhibitor of shoot branching, Teosinte Branched 1/Branched1 (TB1/BRC1), within the bud [165], (ii) the stimulation of the sink strength of the buds for sugars [169,170], and/or (iii) the promotion of auxin export from axillary buds to the main stem [171]. This latter mechanism is considered as a prerequisite for bud outgrowth in *Arabidopsis* and relies on the downregulation of a CK-signaling transcription factor (ARR1) and the upregulation of three auxin efflux carriers (*PIN3*, *4* and *7*) [171,172,173,174]. PIN3/4/7 contributes to the local auxin transport between the PAT stream and surrounding tissues, referred to as connective auxin transport (CAT) [174]. The role of sugars in bud outgrowth is at the core of the historical nutrient diversion theory, which states that bud outgrowth is restricted by competition for the carbon resource in favor of the faster growing apical zone [175,176]. In addition, sugar starvation of the buds is tightly correlated to their dormancy status [163,177]. Sugars act as signaling molecules, as supported by their ability to downregulate *BRC1* [165,178] and by the fact that nonmetabolizable sugar analogs promote bud outgrowth in rose and pea [178,179,180]. Positive systemic signaling associated with sugar has been reported for etiolated stem branching in potato [181]. The T6P signaling pathway takes part in the local and systemic sugar-dependent regulation of bud outgrowth in *Arabidopsis* and in pea [182,183]. All these findings indicate that sugar mediates bud outgrowth through different sugar-signaling pathways, and additional investigations are needed to understand how they drive bud outgrowth individually and/or collectively.

Current evidence shows crosstalk between sugars and SLs in rose, pea and rice [5,184]. In contrast, the basic regulatory mechanisms related to the sugar/CK interplay in the driving of shoot branching is still unknown. Sugars stimulated CK synthesis in one-node cuttings in vitro [178], and CKs could promote the expression of genes associated with the sink strength of buds for sugars [185]. Additional investigations are obviously required to elucidate how sugars and CKs synergistically regulate bud outgrowth. BRC1, which is under the control of sugars and CKs, could be an interesting hub for this regulation (Figure 5) [165].

## 5. Leaf Senescence

Leaf senescence can be a constitutive process of age-related development or an inducible mechanism triggered by unfavorable environmental conditions [186]. During this process, leaf cellular constituents and metabolites are actively recycled and exported to sink organs [22,187,188]. Leaf senescence is driven by sugars and hormones [22,87,187,189,190]. While acting cooperatively in growing leaves, sugars and CKs take on opposite roles in senescing leaves. Sugars promote the appearance of senescence symptoms [191,192]. Glucose highly promotes the expression of *PAP1* and *PAP2*, two senescence-associated MYB transcription factor genes, and of the senescence-specific gene *SAG12* [193]. This sugar-dependent induction of senescence could involve different sugar-related signaling pathways that may work in opposite manners. The best-characterized one is the HXK-dependent signaling pathway: tomato or *Arabidopsis* HXK-overexpressing mutants exhibit high sensitivity to glucose and an accelerated senescence phenotype [194,195], while delayed senescence occurs in HXK knockout mutants [196,197]. Either increased T6P levels or reduced *AtTOR* activity triggers leaf senescence [198,199].

By contrast, CKs delay leaf senescence, as evidenced by results from exogenous supply of CKs, engineered plants with enhanced endogenous CK concentrations, and mutants deficient in CK signaling [200,201,202,203]. However, elevated expression of *IPTs* or LOG7a, two CK-encoding genes, has been unexpectedly reported in detached senescing leaves [43,204,205]. Delayed CK-mediated senescence is dependent on the activity of cell wall invertases (*CWI*) [206]. The tomato mutant deficient in *INVINH1*, an inhibitor of CWI activity– accordingly exhibited low leaf senescence [207]. The role of CWI in retarding leaf senescence is still unclear and may be more related to sugar signaling than to C nutrient provision [208]. CKs could be involved in the regulation of the progression of leaf senescence by ensuring multiple roles, including scavengers of reactive oxygen species (ROS) or the maintenance of mitochondrial integrity [188,201]. Silencing the expression of *RhPR10.1* (pathogenesis-related PR-10) in rose both accelerated flower senescence reduced CK levels and downregulated three CK signaling pathway genes– *RhRR3*, *RhRR8* and *RhRR9* [209]. Additional key components of the CK signaling pathways, including a CK receptor (AHK3), a CK-response factor (CRF6), the type-B response regulator (ARR2) and the CRF-related AP2/ERF transcription factor family, also take part in the senescence-retarding role of CKs [19,210,211].

Although sugars and CKs clearly have opposite effects on leaf senescence, the basic mechanism behind this crosstalk is still mostly unclear. One potential node may be the photosynthetic activity that influences the initiation of leaf senescence [212,213]. Photosynthetic activity is promoted by CKs [214] and repressed by sucrose and other derivative sugars accumulation through HXK-dependent signaling [215,216]. This mechanism involves Abscisic Acid Insensitive4 (ABI4), which encodes an ABA-regulated AP2 domain transcription factor [78]. Involved in the CK-dependent regulation of lateral root development [217], ABI4 may be an integrator of the antagonistic control of leaf senescence by sugars and CKs. Alternatively, a double mutant overexpressing *IPT* and *HXK* also showed early senescence-related characteristics comparatively to the IPT-overexpressing mutant and the WT, indicating a dominant role of sugars in the establishment of leaf senescence [218]. This is consistent with the presence of a set of sugar–signal-related motifs (e.g., YBGAHV, TATCCAOSAMY and ACGTABBOX) in the *Glycine max* promoter region of the *IPT* gene [219]. Future lines of research could target the way IP and root-derived CKs operate to antagonize the inductive effect of sugars on leaf senescence.

## 6. Flowering

**Figure 6:**
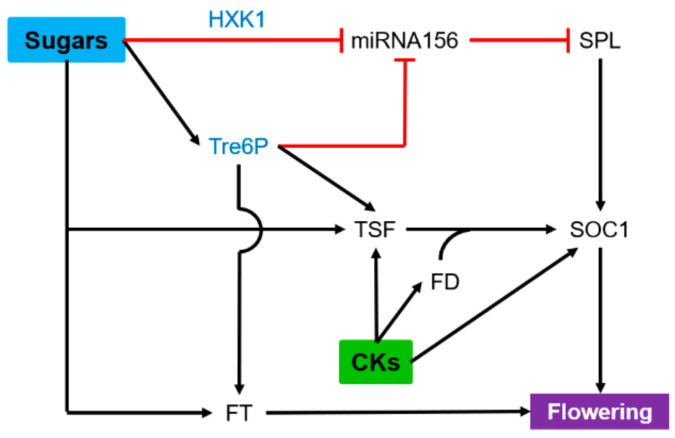
Synergistic effect of sugars and cytokinins (CKs) on flowering. Blue words indicate sugar signaling pathways. Black arrows and red lines indicate stimulatory and inhibitory effects, respectively. FD, FLOWERING LOCUS D; FT, FLOWERING LOCUS T; HXK1, hexokinase1; SOC1, Suppressor of Overexpression of Constans1; SPL, Squamosa Promoter-Binding Protein-Like; Tre6P: trehalose-6-phosphate; TSF, TWIN SISTER OF FT.

Flowering is an important developmental process that ensures plant survival. The transition from the vegetative to the flowering stage must occur in a timely manner to maximize reproductive success. This developmental juvenile-to-adult reproductive switch is controlled by six major regulatory pathways that integrate different environmental and endogenous signals: the photoperiod, vernalization, gibberellins, ambient temperature, autonomous, and age [220]. This flowering network converges toward the major floral integrator gene FLOWERING LOCUS T (FT), its closest homolog TWIN SISTER OF FT (TSF), Suppressor of Overexpression of Constans 1 (SOC1), and FLOWERING LOCUS D (Figure 6) [220,221,222]. In *Arabidopsis*, a facultative long-day plant, sucrose concentrations in leaf exudates increase in response to inductive long days [223]. These increases in sucrose export levels result from carbohydrate mobilization rather than increased photosynthesis [224]. In line with this, sucrose supply can promote flowering in *Arabidopsis* and tomato [225,226]. However, high sucrose concentrations can have an inhibitory effect on floral transition [227]. Besides sucrose, glucose plays a major role in this process through the miR156/SPLs regulatory module identified as a key component of the aging pathway (Figure 6). Thus, the glucose-induced repression of miRNA156 is partly dependent on the signaling activity of HXK1 [228]. Sugar-mediated flower induction may also involve the signaling metabolite T6P, whose accumulation depends on T6P synthase 1 (TPS1) activity [229]. These authors showed that T6P pathway signaling in leaves is essential for both *FT* and *TSF* expression under inductive photoperiod. In addition, the T6P pathway also acts as a local signal in the SAM through the miRNA156-SPLs module independently of the photoperiod pathway. Transgenic *Arabidopsis* plants overexpressing jatropha T6P phosphatase (*JcTPPJ*) display a delayed flowering under inductive long days as compared to the WT [230]. Nevertheless, the *Arabidopsis* knockout mutant *tppi* exhibited the opposite phenotype, i.e., late-flowering under non-inductive conditions. This question means the regulation of flowering time by T6P, but also by downstream products of the T6P pathway like trehalose [231]. TSF inhibits the fructose phosphorylating activity of fructokinase 6 (FRK6) through direct interaction [232]. This potential regulatory role of the TSF-FRKs nexus in determining the flowering time of *Arabidopsis* is supported by the delayed flowering of the *frk6* mutant under short-day conditions. In plants, CKs should be considered as an obligatory component of floral induction and may act both in leaves and shoot apices [233,234]. CK supply to *Arabidopsis* roots indeed promotes flowering and induces transcription of *TSF in leaves as well as FD and SOC1 under short-day conditions* independently of *FT* (Figure 6) [234]. Additionally, exogenous treatment with CKs could also induce *SOC1* in the shoot meristem [233]. Gain-of-function variants of AHK2 and AHK3, two CK receptors, displayed enhanced CK signaling, resulting in early flowering under long-day conditions [235]. Consistent with these results, the rice *hk5 hk6* mutant, disrupted for two HK cytokinin receptor genes, displayed severely delayed flowering [236]. *SOC1*, *FD* and *ARR5*-like were upregulated in sweet cherry tree buds during flowering induction when the highest amount of CKs was applied [237], as they were in apple tree buds when CKs were applied [11].

The role played by the crosstalk between sugars and CKs in the control of flowering is still almost unknown. Additional research is required to evaluate whether TSF, the paralog of FT, could be the main node of the combined effect of T6P and CKs.

## 7. Conclusions

Sugars and CKs play a pivotal role in morphogenesis and plant development because they are predominant during both the vegetative and reproductive stages of plant life (Figure 1). However, the detailed mechanism whereby these two regulators interplay is still puzzling, and many mechanistic scenarios are plausible. Many questions still remain open, include which molecular actors, which hubs could be involved at the crossroads of the sugar and CK signaling pathways. As the sugar/CK interplay can have antagonistic or agonistic outcomes, its regulatory network is expected to be complex and multifactorial depending on developmental and environmental inputs. Sugars and CKs both regulate the relationships between source and sink organs at the whole plant level. As a consequence, we may wonder about the relevance of the main energy and nutrient status sensors (Sucrose non-fermenting-related kinase (SnRK1)/target of rapamycin (TOR kinase)) in this process. The involvement of these mechanisms in this crosstalk deserves to be investigated. Meanwhile, our knowledge about the roles of sugars and CKs in the plant response to stressors is well investigated, but data about their crosstalk is again still very limited. Such an understanding is crucial to building up a comprehensive picture in different biological contexts throughout plant life. Further works are thus needed to fully investigate the regulatory networks behind the crosstalk between sugars and CKs. This will undoubtedly help to suitably manage plant physiology in view of increasing agronomy and resilience performances in an ever-changing environment.

## Data Availability

Not applicable.
